# Introduced species that overcome life history tradeoffs can cause native extinctions

**DOI:** 10.1038/s41467-018-04491-3

**Published:** 2018-05-30

**Authors:** Jane A. Catford, Michael Bode, David Tilman

**Affiliations:** 1 Biological Sciences, University of Southampton, Southampton, SO17 1BJ UK; 2 School of BioSciences, The University of Melbourne, VIC, 3010 Australia; 3 Fenner School of Environment and Society, The Australian National University, Canberra, ACT 2601 Australia; 4 Department of Ecology, Evolution and Behavior, University of Minnesota, Saint Paul, MN 55108 USA; 5 School of Mathematical Sciences, Queensland University of Technology, Brisbane, QLD 4001 Australia; 6 Bren School of the Environment, University of California, Santa Barbara, CA 93106 USA

## Abstract

Introduced species threaten native biodiversity, but whether exotic species can competitively displace native species remains contested. Building on theory that predicts multi-species coexistence based on a competition-colonisation tradeoff, we derive a mechanistic basis by which human-mediated species invasions could cause extinctions through competitive displacement. In contrast to past invasions, humans principally introduce modern invaders, repeatedly and in large quantities, and in ways that can facilitate release from enemies and competitors. Associated increases in exotic species’ propagule rain, survival and competitive ability could enable some introduced species to overcome the tradeoffs that constrain all other species. Using evidence from metacommunity models, we show how species introductions could disrupt species coexistence, generating extinction debts, especially when combined with other forms of anthropogenic environmental change. Even though competing species have typically coexisted following past biogeographic migrations, the multiplicity and interactive impacts of today’s threats could change some exotic species into agents of extinction.

## Abstract (web summary)

Introduced species may displace ecologically similar native species, but mechanisms are still to be established. Here, Catford et al. provide theoretical evidence of how human-mediated species invasions may overcome competition-colonisation tradeoffs, leading to the local extinction of native species.

## Introduction

Introduced species are documented as threatening native biodiversity^1,2^, but whether exotic species can competitively displace ecologically similar native species remains contested^3–5^. This debate has been fuelled by a dearth of identified causal mechanisms^6^, inconsistent relationships observed between invasion and diversity^7–9^, and evidence from the fossil record where prehistoric mass species incursions caused few, if any, extinctions through competition^10^. In this paper, we use a theory that predicts species coexistence based on interspecific tradeoffs^11^ to derive a mechanistic basis by which human-mediated species invasions could cause extinctions through competitive displacement.

Interspecific (or life history) tradeoffs offer a leading explanation for multi-species coexistence^11,12^: if each species (regardless of origin) occupies a unique position along the same multi-dimensional competitive tradeoff surface, then an invading species cannot competitively displace any existing species because no species can outperform others under all conditions^10^. This “universal tradeoff hypothesis” asserts that traits of ecologically similar species are bound to the same interspecific tradeoff surfaces, regardless of species’ biogeographic or phylogenetic origins^10^ (but see ref. ^13^). A key corollary of this hypothesis is that invading species should stably coexist with ecologically similar native species in spatially heterogeneous habitats, a prediction supported by palaeontological studies examining effects of past species migrations to new continents^10^. If universal tradeoffs exist, such that all species are bound by the same rules regardless of their origin, then any conditions that allow invading species to overcome such tradeoffs could result in extinctions of competing species.

Focusing on the well-known competition-colonisation tradeoff, whereby an increased allocation towards reproduction and dispersal comes at a cost of species’ ability to compete for a limiting resource^11^, we consider two processes that are key features of modern invasions—cultivation and enemy release—and use them to highlight ways that human introduction could make modern invasions qualitatively distinct from those of the past. Human-influenced processes, like the two we examine, could allow some modern invaders to move off the competition-colonisation tradeoff surface, potentially resulting in local extinctions of native species. We note, though, that most introduced species do not become dominant, displace native species or experience increased abundances in their introduced ranges^14,15^, and many fail to even establish outside of areas where they are cultivated^16,17^.

First, humans introduce modern invaders, usually deliberately^18^, and sometimes in very high numbers over broad spatial and temporal scales. Pasture for livestock production is the greatest land use on Earth, covering 30% of the global land surface^19^. Over 90% of plant taxa developed and sold by agribusinesses are known to invade native ecosystems somewhere in the world, and on average 30% of pasture plants are exotic and invasive in the country in which they are sold^20^. Exotic species that become invasive tend to be introduced more often and over longer periods of time than exotic species that do not become invasive^18^. Higher numbers of propagules can increase the probability of species establishment in a new environment^21^ and may increase the local abundance of seed-limited species^22–25^ (though not always^16^). In the fossil record, dispersal and migration of invaders was reliant on invaders’ own colonisation abilities, which were limited through tradeoffs. Today, humans plant and disperse some species in high numbers, increasing the colonisation rates of some modern invaders in a way that is independent of their ecophysiological traits^26^. For example, propagules of the exotic ornamental herb, *Ruellia simplex* (Mexican petunia), planted in private and commercial gardens in Florida, disperse in stormwater runoff to nearby floodplain forests; there they supplement the local seed supply, creating and maintaining *R. simplex* monocultures that displace native plant species^27^. Although the vast majority of exotic species would not experience sustained, or ecologically meaningful, external propagule inputs, pasture plants are often repeatedly planted across broad areas of the landscape^20^, and other exotic plants are widely used in gardens, horticulture, agriculture and silviculture. As such, there is a greater (and demonstrated^28,29^) risk that widely cultivated exotic plants will invade remnant native vegetation nearby, potentially threatening native species.

Second, humans can release some modern invaders from their enemies (and competitors) by deliberately importing pest- and disease-free individuals for use in cultivation, and by rapidly transporting species beyond their historical biogeographic boundaries^30,31^. Across 473 European plant species recently naturalised in the US, for example, an average of 84% fewer fungi and 24% fewer viruses infect each species in their introduced range than in their native range, and the species with greater pathogen release were more commonly listed as noxious and invasive^32^. In the past, enemies were more able to move and migrate with their hosts, and the absence of human transport made the invasion process more gradual. Slower rates of invasion would have given local predators, pathogens, parasites, herbivores and competitors time to adapt to (and thus start limiting) invaders before the invaders were able to completely displace native species. Today that is not necessarily the case; *Alliaria petiolata, Microstegium vimineum* and *Berberis thunbergii*, three exotic plant species that are currently invasive in the US, are indirectly facilitated by a native generalist herbivore, the white-tailed deer, which preferentially grazes co-occurring native plants^33^ (but see ref. ^34^). Not all exotic species introduced by humans experience beneficial enemy release^35–38^ or experience it forever^39,40^, but those that do experience it can potentially: live longer^41^, grow larger^42^, reach higher abundances^33^, expand their environmental range^41,43^, increase their competitive ability^44^, reproduce more^42^, and reproduce more successfully^45^ in their introduced ranges than in their native ranges.

**Fig. 1:**
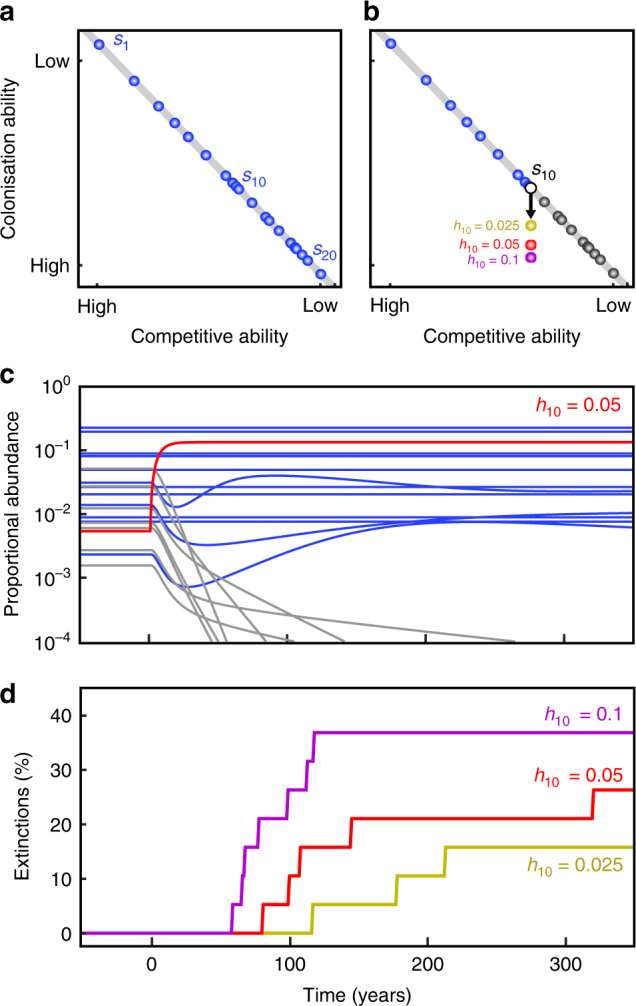
Simulated species extinctions resulting from human-mediated species invasions. **a** Competition-colonisation tradeoff surface (grey line) of 20 coexisting species (blue circles) in a metacommunity, going from the best competitor (*s*_1_) to the worst competitor (*s*_20_). Species’ competitive ranks are inverse to the rank order of species’ colonisation abilities (this is the tradeoff). Note that, regardless of positions on the *X*-axis, differences in species’ competitive abilities are determined by rank only such that the competitive difference between *s*_1_ and *s*_2_ is the same as the competitive difference between *s*_18_ and *s*_19_. **b** Schematic drawing of disrupted tradeoff showing shifted position of designated “invader” (*s*_10_, the intermediate competitor in the metacommunity, white circle indicates *h*_10_ = 0) reflecting input of external colonists following Eq. 1 (i.e. increasing *h*_10_ from 0 to 0.025 (yellow), 0.05 (red) and 0.1 (violet); shifted positions of *s*_10_ on the figure were chosen to reflect the number of observed extinctions: 3 for *h*_10_ = 0.025, 5 for *h*_10_ = 0.05 and 7 for *h*_10_ = 0.1); blue circles indicate “native” species that are superior competitors and thus unaffected by the invasion; grey circles indicate the ten natives that could be displaced by *s*_10_. **c** Relative abundance (log scale) of each of the 20 species over time. Each line represents a species: the red line indicates the invader after elevating *h*_10_ to 0.05; blue lines indicate persistent native species; grey lines indicate native species driven to extinction (i.e. relative abundance drops below 0.0001 of species’ original equilibrium abundance) as a consequence of increasing *h*_10_. **d** A timeline of percentage of the 19 native species driven to extinction as a consequence of elevating *h*_10_ to 0.025, 0.05 (same scenario shown in **c**) and 0.1

These two processes, which stem from human cultivation and the novel evolutionary histories of exotic species, could effectively move some exotic species off a universal tradeoff surface by enabling them to become better colonisers, better competitors or longer-lived than their native counterparts (Fig. 1a, b) (Supplementary Table 1). Critically, we note that these are not the only processes that could enable species—and exotic species in particular—to move off interspecific tradeoff surfaces^31^ (Supplementary Table 2); we simply use these two prominent, and mechanistically distinct, hypotheses to illustrate our theory. Reduction of natural enemies is likely to be particularly powerful in overcoming tradeoffs since it can collectively lower mortality rates, enable plants to grow faster and larger and thus produce more seed, and increase species’ competitive abilities by allowing them to live on lower levels of limiting resources^39^. This combination of decreased mortality, higher seed production and increased competitive ability pose a triple threat of extinction, and—unlike effects of external propagule inputs, which are local—enemy release could occur across an entire area that is invaded. The impacts of mass cultivation and enemy release on diversity may be exacerbated because today’s species introductions coincide with other forms of anthropogenic global change (Supplementary Table 2). Habitat loss, nitrogen deposition, climate change and changes in disturbance regimes can threaten native species directly, but they can also augment the benefits of high propagule rain^25,26^ and enemy release^33,46^ to exacerbate invasions (though not always^22^).

In this paper, we use theoretical evidence to determine the conditions for which an exotic species could plausibly lead to local extinctions of its native competitors. We first present a model that illustrates how competition-colonisation tradeoffs allow species to coexist^11,47^. We expand the model to incorporate ways in which modern species invasions may disrupt this mechanism of species coexistence, even when the invaders themselves are intrinsically bound by universal tradeoffs. We then analyse ensembles of metacommunity models to identify ways in which human-mediated species invasions, alone and with elevated disturbance, could plausibly drive one or more native species extinct. Our theory predicts that: i) human-mediated species invasion—here characterised by enemy release and input of external propagules—can eventually drive ecologically similar native species locally extinct; ii) predicted extinctions are preceded by gradual changes in species’ relative abundances and are dependent on the characteristics of the invader relative to the native community; and iii) extinctions are most likely when invasion co-occurs with other forms of environmental change. Competing species have coexisted for millions of years following past biogeographic migrations^10^. However, the multiplicity and interactive impacts of today’s threats may cause some exotic species to become agents of extinction.

## Results

### Model framework

In the coexistence model based on a competition-colonisation tradeoff that we use, species are ranked from the best to the poorest competitor (*s*_1_ to *s*_*N*_ in an *N*-species metacommunity)^11^. Species are able to coexist if their colonisation abilities are inversely related to their competitive rank (Fig. 1a). The model assumes a perfect competitive hierarchy where superior competitors can displace inferior competitors, and the weakest competitors only colonise vacant sites. The proportion of sites, *p*_*i*_, occupied by species *i* over time, *t*, is therefore determined by species *i’*s per-capita colonisation rate, *c*_*i*_, the introduction of its propagules from an external source, *h*_*i*_ (which can be temporary or sustained), its per-capita mortality rate, *m*_*i*_, and its competitive rank, *i*, where a species with a lower *i* is competitively superior to all species with higher *i* (i.e. *j* is competitively superior to *i*; see Methods):

1 $$\frac{{{\rm d}p_i}}{{{\rm d}t}} = \left( {c_ip_i + h_i} \right)\left( {1 - \mathop {\sum }\limits_{j = 1}^i p_j} \right) - \left( {m_i + \mathop {\sum }\limits_{j = 1}^{i - 1} \left( {c_jp_j + h_j} \right)} \right)p_i.$$

### Species cultivation

The colonisation rate of a “natural” species is proportional to its population size (via *c*_*i*_*p*_*i*_ in Eq. 1), but species deliberately planted in gardens, silviculture, horticulture and agriculture, which make up the majority of invasive plants^18^, can have supplies of externally sourced propagules that are independent of their local abundance (*h*_*i*_ in Eq. 1). Native species may also receive external inputs of propagules if they are planted and cultivated by humans. However, modern exotic species are more likely to experience increased *h*_*i*_ because, by definition, they are introduced by humans and associated with humans.

**Fig. 2:**
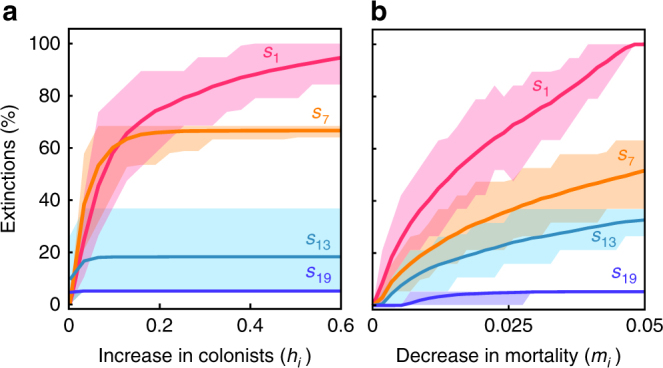
Native species extinctions resulting from species invasion in 20-species metacommunities where the invader experiences an increase in external colonists (*h*_*i*_), and a reduction in mortality (*m*_*i*_). For each line in each panel, a new species has been introduced that would normally coexist with the 19 other species in the metacommunity given their natural colonisation and competitive abilities (i.e. the invader naturally falls upon the universal tradeoff surface). However, input of external colonists (*h*_*i*_, shown in panel **a**) and reduced mortality (*m*_*i*_, shown in panel **b**) (changes in *h*_*i*_ and *m*_*i*_ are both measured on the *X*-axis) of the invader allows it to overcome this tradeoff and cause extinctions. Where lines plateau, all inferior competitors have been driven to extinction. The competitive rank of the invaders is indicated by different coloured lines: *s*_1_ = pink (superior competitor), *s*_7_ = orange, *s*_13_ = light blue, *s*_19_ = dark blue. Lines indicate average effects across an ensemble of 250 metacommunities; shaded areas enclose 95% of the metacommunity responses

We find that the addition of an external propagule supply (*h*_*i*_) increases the relative abundance of an invader (*s*_10_ in Fig. 1), which can subsequently exclude or reduce the abundance of some or all of the species that are its inferior competitors (Fig. 1, Supplementary Figs. 1–3), a result that is consistent across simulations of 250 different 20-species communities (Fig. 2a). The probability and extent of species displacement increases over time as the population size of the invader increases, and effects cascade through the populations of inferior competitors (Fig. 1). Generally, the further a species is shifted off the tradeoff surface through higher values of *h*_*i*_, and the greater the number of (ecologically similar) inferior competitors the invader has, the higher the rate and number of extinctions that eventually occur (Fig. 1, Supplementary Figs. 1, 2). Native species that have similar colonisation abilities as the invader (but are competitively inferior), and are thus positioned close to the invader on the (undisrupted) tradeoff surface, are most affected by additions of *h*_*i*_.

Temporary (as opposed to permanent) increases in *h*_*i*_ cause transient reductions in species’ abundances that remain visible many generations after the introduction event (Supplementary Fig. 3). Although most populations may ultimately recover, extended periods of low abundance heighten species’ exposure to demographic and environmental extinction risk^6^, so even temporary increases in invader *h*_*i*_ may threaten native species persistence.

### Enemy release

Species may experience lower rates of enemy attack, including seed predation, when introduced beyond their historic range^30^. Rather than discussing the generality of enemy release^48,49^ (and other hypotheses that invoke the novel evolutionary histories of exotic species, which may shift species off the tradeoff surface through changes in biotic interactions^26,31,50^), here we focus on the potential implications of enemy release—if and when it occurs—on species coexistence.

We model the direct effects of enemy release by reducing the mortality rate (*m*_*i*_) of individual invasive species, but recognise that enemy release may also increase a species’ colonisation ability (*c*_*i*_) and its competitive rank^30,42,44^. As with increased *h*_*i*_, decreased *m*_*i*_ causes the abundance of the released invader to increase to the detriment of inferior competitors, which may be driven extinct (Fig. 2b). We note that direct increases in species’ competitive ability, which would accompany reductions in mortality and tissue loss (Supplementary Fig. 4)^44^, could also cause competitive displacements following enemy release. Among other examples^26,51,52^ (Supplementary Table 1), observations of a doubled growth rate and greater shade tolerance, as well as 41% lower mortality, of the invasive shrub *Clidemia hirta* when released from fungal and insect enemies seem to support this prediction^41^. The greatest number of extinctions, of course, would come from an invader having: more propagules; decreased mortality; and increased competitive ability.

### Environmental change

Modern biological invasions occur in the context of multiple anthropogenic environmental changes, including climate change, habitat destruction, habitat fragmentation, nutrient deposition and altered disturbance regimes (Supplementary Table 2), each of which could reduce the abundance of one or more native species. Such reduced abundances might, in combination with human-mediated invasions, cause extinction even if neither factor would do so on its own.

**Fig. 3:**
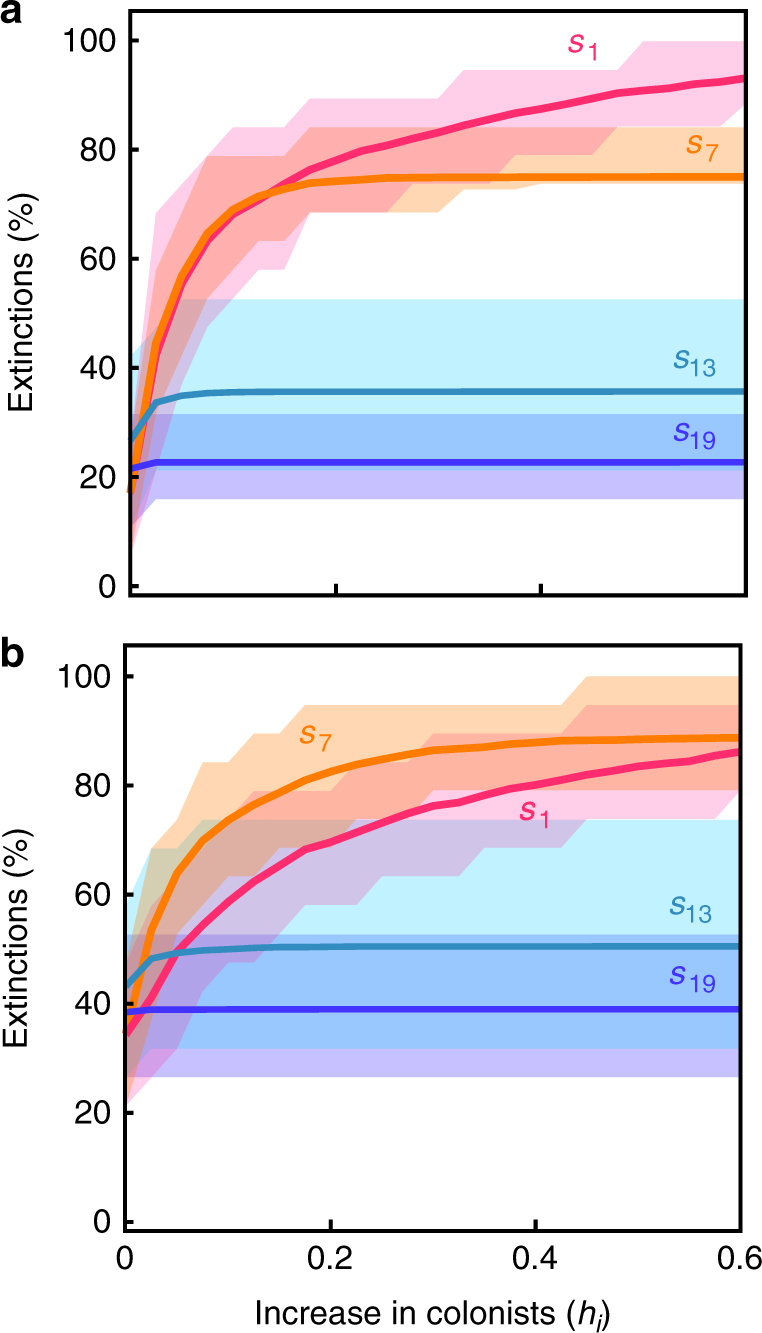
Native species extinctions resulting from elevated disturbance and invasion of a species with a supply of external colonists (*h*_*i*_). Disturbance increases the mortality rate of all 20 species in the metacommunity from 0.05 to **a** 0.06, and **b** 0.35. Other details as in Fig. 2. In all panels, species are lost from the metacommunity due to increased disturbance alone (i.e. when *h*_*i*_ = 0). Invasion of *s*_7_ causes more extinctions than *s*_1_ under high disturbance (shown in **b**) because the natural colonisation rate of *s*_1_ (*c*_1_) is too low to cope with elevated mortality, unless increases in *h*_1_ compensate for it

To gauge potential interactive effects of invasions and environmental change, we examined diversity consequences of species-specific increases in *h*_*i*_ when they are set against a backdrop of elevated disturbance (increasing *m*_*i*_ of all species in the metacommunity), as would be experienced with increased trampling, mowing, storm damage, fire, and so on. Increasing mortality rates (*m*_*i*_) of all species generally heightened the number and rate of extinctions caused by species-specific increases in *h*_*i*_ (Fig. 2a cf. Fig. 3). By itself, elevated disturbance also led to species losses. As with the specific example of habitat destruction^53^, elevated disturbance disproportionately reduces the abundance of poor colonisers (which are also better competitors, reflecting tradeoffs), increasing their risk of extinction. Elevated disturbance correspondingly limits the impact of competitive invaders, which are poor colonisers (*s*_*1*_ in Fig. 3). Species-specific increases in *h*_*i*_ and reductions in *m*_*i*_ can counteract effects of elevated disturbance, meaning invaders would be less affected by disturbance than native species, all else being equal.

## Discussion

About 13,000 vascular plant species, 4% of the world’s flora, are naturalised outside of their native range^54^. Each of these exotic species must allocate some of its resources to disperse, establish and reproduce—just like native species. However, human intervention means that exotics may not experience the full suite of constraints experienced by native species in vicinities where they are introduced and cultivated^26^.

Compounding the introduction and dispersal advantage of cultivated plants, laws of many countries only allow the importation of exotic plants if they are certifiably free of pests or pathogens, resulting in a joint *h*_*i*_ and *m*_*i*_ scenario. This biosecurity requirement is designed to prevent the arrival of new pests and pathogens that could devastate agriculture, horticulture and forestry industries (e.g. Dutch elm disease, *Phytophera)*. However, exotic species that had been particularly strongly restrained in their native habitat by high enemy loads may therefore be unusually successful in habitats in which they become essentially enemy-free (Supplementary Table 1). This dynamic may partially explain why some species with highly restricted native ranges can become globally invasive. For example, Monterey pine (*Pinus radiata*) is planted in over 4 million hectares throughout the world for timber production, and is the dominant tree plantation species used in Chile, Australia and New Zealand^55^. It has escaped from cultivation and invades native vegetation, and is a threat to biodiversity  in much of the Southern hemisphere^56^ where its invasiveness is partially attributed to enemy release^55^. *P. radiata* has a tiny native range in California and Mexico where it naturally experiences high mortality from frequent wildfires, is the primary host of a mistletoe parasite, and suffers high disease loads^55,57^.

Species that are rare in their native ranges but highly invasive elsewhere, like Monterey pine, Cootamundra wattle (*Acacia baileyana*), Blue gum (*Eucalyptus globulus*), Small balsam (*Impatiens parviflora*) and Yellow start thistle (*Centaurea solstitialis)*, may exemplify species that have overcome an interspecific tradeoff because of human introduction. Demographic data from 625 plant species suggest that invasive species may flout the fast growth versus long survival tradeoff that constrains other species because, unlike the vast majority of non-invasive species, they are able to achieve high reproduction and fast growth rates without compromising survival^51^. How these species might be overcoming this tradeoff is largely unexplored, but there is some suggestive evidence that enemy release could be responsible. The ability of *Acer platanoides*, an invasive tree in the US, to maintain high growth rates under both high and low light^43^ has been attributed to it experiencing a three-fold reduction in herbivory in its introduced versus native range^58^, and lower herbivore attack than a native congener^59^. Similar observations have been made in tropical^41^ and temperate^25^ forests for other exotic species (Supplementary Table 1). The notable success of some biocontrol agents in constraining deliberately introduced exotic species, like Prickly pear (*Opuntia stricta*), *Salvinia molesta* and Gorse (*Ulex europaeus*), demonstrates the importance of confining invaders to their position on interspecific tradeoff surfaces for species coexistence.

Our theoretical model predicts that invading species that overcome universal tradeoffs could eventually displace co-occurring native species that occupy similar niches as the invader. [If using Chesson’s framework^60^, this can be conceived as enhancing the fitness of invaders relative to co-occurring natives^61^]. Local extinctions of native species have been observed that may stem from the disruption of interspecific tradeoffs (whether via human introduction, enemy release or another mechanism^31^, Supplementary Table 3), but such extinctions are generally predicted to occur tens to hundreds of generations after the onset of species invasion^10^. The ability to overcome interspecific tradeoffs is not necessarily restricted to exotic species (though exotics are much more likely to experience enemy release and other benefits of novel evolutionary histories, in particular, than natives), and likely applies to more than just plants. Patagonian lakes with higher aquaculture intensity have higher abundances of exotic salmonids and lower abundances of native fish^62^, and exotic populations of animals have half as many parasite species and experience lower infection rates per individual than native populations^63^. Experiments designed to directly test this theory will no doubt be very informative.

By identifying mechanisms through which exotic species introduction and cultivation could prompt the local extinction of native con-trophic species, our theory may help resolve a question that has inspired vigorous debate in ecology and conservation biology^3–5^. The theory predicts that invasion-induced con-trophic extinctions are local, delayed, preceded by gradual changes in species’ relative abundances, dependent on the characteristics of the invader relative to the native species, and are most likely when invasion co-occurs with other forms of global change. These conditions are matched by empirical observations^2,26^. When considering the two processes that we have focused on—cultivation and enemy release—impacts are likely to be greatest when the invaders are highly competitive yet ecologically similar to native species (akin to large fitness but small niche differences^60,61^), intensively cultivated or planted, and when they experience high levels of enemy release. Many species introduced to date may be poor competitors^64^, and only some of these species are likely to have been moved off the tradeoff surface far enough, or for long enough, to meaningfully reduce the abundances of native species. So, while global extinctions are a possible outcome of our proposed mechanism, they have, at least for now, been relatively rare^2^.

We appreciate that our theoretical assumptions simplify the real world, and do not suggest that all exotic species will overcome the tradeoff surface or will cause native extinctions. Rather, we illustrate a mechanism by which species invasion, and particularly the introduction of enemy-free species and mass cultivation, could cause the eventual displacement of ecologically similar native species. Given that species displacement occurs over many generations (Fig. 1, Supplementary Figs. 1, 2)^6,10^, and that the majority of species introductions have occurred in the last 200 years and at rapidly increasing rates^65^, it seems plausible that most invasion-induced extinctions are yet to occur.

## Methods

### Overall approach

We used ensembles of multispecies metacommunity competition models to simulate the dynamics of communities where one species does not conform to a competition-colonisation tradeoff^11,66^.

### Generating an ensemble of stable metacommunities

The metacommunity model was continuous in space, with the abundance dynamics of each population described by the proportion of the total available area they occupy (Eq. 1)^11,66^. Equilibrium abundances for 20-species metacommunities were found by iteratively solving the equations, from the most competitive to the least competitive species. To robustly assess the effect of invaders on extinction rates, we generated a large ensemble of different metacommunities, each with 20 coexisting species. Each species’ colonisation rate was chosen independently from a uniform random distribution *c*_*i*_ *~ U*(0,5), and their competitive ranking was assigned as the inverse of their colonisation ranking. All species had the same mortality rate of *m* = 0.05. The input of external propagules was initially set to zero (*h*_*i*_ = 0). A search of 5 × 10^8^ randomly generated metacommunities yielded 250 that could stably coexist, and the following analyses and conclusions are based on this entire ensemble.

### Assessing effects of invaders overcoming a tradeoff surface

Using the stable 20-species metacommunities created as outlined above, we designated one of the coexisting species, *a*, as the “invader”, and manipulated its propagule supply (*h*_*a*_) and mortality (*m*_*a*_) such that it could overcome the competition-colonisation tradeoff. We used this approach, rather than adding an additional species, to ensure we were using a combination of species that would ordinarily coexist, i.e. without human intervention. We increased *h*_*a*_ to simulate an increase in the invader’s propagule rain (which affects net colonisation). The scale of the variable *h*_*i*_ can be understood in relation to the proportion of available microsites (the space required for an individual plant); for example, a value of *h*_*i*_ = 0.1 implies that propagules from anthropogenic sources would be sufficient to colonise 10% of microsites in an entirely vacant plot. We reduced, *m*_*a*_, the mortality of the invader, to simulate enemy release. When we simulated elevated disturbance, we increased the value of *m*_*i*_ for all species in the metacommunity (including the invader) by the same amount.

For each analysis, we designated a particular species to be the invader, and simulated the resulting changes in the metacommunity abundances. Figure 1c shows the resulting changes in the relative abundance of each species through time, *p*_*i*_ (*t*), for a single metacommunity. Species extinctions were calculated as the proportion of the 19 “native” species that had been lost from the metacommunity at a given time. We defined a species as extinct at time *t* if its abundance declined below 0.01% of its original equilibrium abundance at any time prior to *t*.

### Model complexity

Although the competition-colonisation tradeoff model has been extended and developed^53,67,68^, we use a simple version to ensure that our points are clear^69^, but note that our modifications could be applied to all variants of the model.

In particular, we used a limited number of scenarios and a simple theoretical model that (i) does not include niche preemption, (ii) has uniform mortality across all species, and (iii) specifies that the release of invaders from natural enemies only results in a reduction in mortality. If we had used replacement rather than displacement competition (i.e. allowing for niche preemption by waiting for individuals to die before microsites can be recolonised^70^), the relative advantage of *h*_*i*_ would have been greater, potentially enabling poorer competitors to exclude better ones^67^, especially when combined with elevated disturbance. If we had allowed mortality rates to vary among species such that mortality was part of the tradeoff enabling coexistence (i.e. a three-way tradeoff among species’ colonisation ability, competitive ability and mortality), elevated disturbance would be expected to have disproportional impact on species that usually rely on low rates of mortality as a mechanism for coexistence.

Our model used a 20-species metacommunity positioned along one tradeoff surface and assumed that there was complete overlap in spatial and temporal patterns of species’ growth (e.g. no effect of varying phenology, growth form or architecture, which are known to be important^24^). This is a necessary simplification of naturally occurring communities, and we acknowledge that the predictive power or applicability to a specific system can be limited with such a simple approach^71^. We nevertheless contend that our key points, demonstrated here, apply to more complex systems and more diverse communities because of the role of interspecific tradeoffs in facilitating species coexistence. Species coexist via multiple tradeoffs, reflecting the myriad resources for which species can compete (e.g. light, nutrients, water)^12^. The ideas we have discussed in our paper should be applicable to these tradeoffs.

### Code availability

Results can be reproduced from code available on GitHub: https://github.com/MikeBode/Introduced_Tradeoffs.

### Data availability

Results can be reproduced from the models described in the paper, but these data are also available from the corresponding author upon reasonable request.

## Electronic supplementary material

Supplementary Information
