# Supplementary material for: Introduced species that overcome life history tradeoffs can cause native extinctions
Source: Nat Commun. 2018 May 30;9:2131. doi: 10.1038/s41467-018-04491-3 (PMC5976637; doi:10.1038/s41467-018-04491-3)
Supplement: Supplementary file 1 — Supplementary Information [file 41467_2018_4491_MOESM1_ESM.pdf]

Introduced species that overcome life history tradeoffs can cause native extinctions

Catford et al.

Supplementary Information

Supplementary Table 1: Exotic plant species that appear to overcome some interspecific life history tradeoffs. Modified from[1].

Exotic species	Ecosystem	Likely mechanism for overcoming tradeoff	Description and life history characteristics
17 invasive species	Various	Not examined	Demographic characteristics of 17 species classified as invasive (vs 603 species classified as non-invasive) suggest that some invasives can violate life history tradeoffs that constrain native species; invasives were more likely than natives to be long lived, with fast leaf economics and high reproduction[2].
169 species from Asteraceae, Brassicaceae, Fabaceae and Poaceae	California	Not examined	Random subset of 831 native and 169 exotic species from four families in California revealed that there was a positive correlation between seed size and plant height in natives but not exotics, suggesting that exotics may overcome the competition–colonization tradeoff by maintaining high dispersal ability even in large sized plants[3].
30 exotic understory	Common garden, temperate deciduous	Greater N foraging ability of exotics, possibly coupled with	Series of studies of up to 43 native and 30 exotic understory woody species revealed that exotics generally exhibit a longer

deciduous woody species	forests, NE USA	increased atmospheric N deposition and invasion of exotic earthworms that stimulate nitrification.	growing season, faster growth and assimilation, and lower nutrient conservation, but also have longer leaf lifespan and high shade tolerance compared to natives[4,5]. Exotics have smaller genome size, which allows faster growth[6].
Acer platanoides (tree)	Forests, USA	Enemy release	High physiological plasticity and shade tolerance; can maintain high growth rates under both high and low light[7,8].
Ageratina adenophora (shrub)	Common garden	Release from generalist and specialist enemies	Allocates more nitrogen to photosynthesis (growth) and less to defense (cell walls) in its introduced range of China and India than in its native range of Mexico, resulting in increased growth rates[9].
Clidemia hirta (shrub)	Forest understorey, Hawaii	Enemy release	Release from fungal and insect enemies results in a doubled growth rate, greater shade tolerance, and 41% lower mortality[10].
Ligustrum robustrum subsp. *walkeri* (tree)	Wet forests, La Réunion	Primary mechanism unknown, but facilitated by human disturbance	Shade tolerance, rapid growth, high seed production, bird-assisted seed dispersal and high seedling recruitment enables *L. robustrum* to form dense, monotypic thickets[11].

Miconia calvescens (tree)	Wet forests, Tahiti	Enemy release	Shade tolerance, wide range of germination conditions, ability to resprout and ruderal characteristics, e.g. fast growth rates, prolific seed production, efficient dispersal, early reproductive maturity. Introduced for botanic garden[12].
Microstegium vimineum (grass)	Forest understoreys, USA	Enemy release	High propagule pressure, high physiological plasticity and shade tolerance; important interaction between propagule pressure and canopy disturbance[13,14].
Pittosporum undulatum (tree)	Wet forests, Jamaica	Enemy release	Displays high physiological plasticity, low mortality, shade tolerance and rapid growth. Trunk growth rates 4-5 times higher than co-occurring natives; as its basal area increased, the basal area of native species and stand-level diversity declined. Lower rates of herbivory and disease likely contributed to high growth rates and survival (zero tree mortality observed during 14-year study). There was a concerted introduction effort of *P. undulatum* at nearby botanic garden, and evidence for allelopathy as well as enemy release[15].

Jacobaea vulgaris (forb)	North America, Australia, and New Zealand	Release from specialist enemies	Absence of specialist herbivores in invasive populations resulted in the evolution of lower protection against specialists and increased growth and reproduction, but also allowed a shift towards higher protection against generalist herbivores[16].

Supplementary Table 2: Some ways in which human activities and global environmental change can exacerbate invasion impacts and species extinctions. These environmental changes may modify conditions that affect multi-species coexistence (i.e. by altering parameters in Eq. 1 in main paper). Many of these environmental changes co-occur (e.g. nitrogen deposition, elevated disturbance, introduction of species that are good colonisers), compounding their individual effects on native diversity, as indicated by Fig. 3 in main paper. NB. This list is not exhaustive, but is used for illustrative purposes.

Parameter	Modification processes	Implications
Population sizes (p_i)	Changes in response to other parameters in Eq. 1.	Increases in p_i increase self-generated seed supply through relationship with c_i and reduces space for colonisation by inferior competitors $(1 - p_j)$.
Colonisation rates (c_i)	Species-specific:<br>Selection[17], breeding[18] and introduction[19] of species with high fecundity and high growth rates can lead to invaders with high c_i.<br>Through reductions in seed predation, enemy release can directly increase c_i.<br>Through reallocation of resources previously used for defense[16], enemy release can indirectly	Increased population sizes; displacement of inferior competitors; potential exclusion of superior competitors if niche preemption occurs[21].

	increase c_i through e.g. increased fecundity, faster growth and reproduction rates.<br>Community-wide:<br>Human vectors and transport networks can increase the dispersal of certain species[20].	
Externally sourced propagules (h_i)	Species-specific:<br>Humans deliberately or accidentally introduce propagules from external sources (e.g. for aquaculture[22], recreational hunting, gardening, pasture production[18], ballast, stowaways[23]).	An external source of propagules can inflate species' population sizes, and allow species to persist despite low population viability (i.e. h_i is independent of p_i), affecting competition among species and open space.
Space for colonisation ($1 - p_j$)	Species-specific:<br>Changes in population sizes of superior competitors (p_i) increases or decreases space available for inferior competitors; if niche preemption occurs, changes in population sizes of any species could potentially affect colonisation success of all other	Lower population sizes of superior competitors provides more space for inferior competitors, resulting in increased dominance of colonisers[24].

	species[21].	
	Community-wide:	
	Habitat destruction and land use change can reduce available space for species' populations, effectively reducing $1 - p_j$ by reducing the total space available for organisms to grow.	Species with low colonisation rates unable to maintain populations of a size that are sufficient to produce enough colonists to ensure the species' ongoing persistence[24] (s_1 in Fig. 3b).
Mortality rates (m_i)	Species-specific:	
	Any amount of enemy release can reduce species' rates of tissue loss and mortality.	Reductions in tissue loss and mortality can increase the survival (and thus p_i)[10] and competitive ability of species[25] (Supplementary Fig. 4).
	Community-wide:	
	Elevated disturbance reduce can reduces species' population sizes (p_i).	Species with low colonisation rates may be unable to keep pace with elevated mortality ($m_i > c_i$), resulting in their loss from the community (s_1 in Fig. 3b).
		Reduced abundance of competitors and increased space for colonisation ($1 - p_j$), favours species with high colonisation rates, facilitating their dominance[24].
Competitive	Species-specific:	

rank	Reduction in tissue loss directly increases species' competitive ability[25] (Supplementary Fig. 4).<br><br>Enemy release can lead to reallocation of resources from defense to enhanced competitive ability[16].<br><br>Novel allelopathic chemicals may increase invaders' relative competitive ability[26,27].	Increases in competitive ability may allow invaders to move up the competitive hierarchy without a reduction in their colonisation ability, thereby moving them off the tradeoff surface.
	Community-wide:	
	Changes in environmental conditions that increase resource availability can reduce niche dimensionality and the relative advantage of high competitive ability, effectively equalizing species' competitive ranks.	Superior competitors lose their advantage of being able to successfully compete for a limited resource, effectively flattening the tradeoff surface such that it becomes one-dimensional and colonisation ability becomes paramount[28,29]. With reduced niche dimensionality, fewer species will coexist, and (formerly) superior competitors will be displaced by superior colonisers.

Supplementary Table 3: Examples of exotic plant species that displace one or more native species resulting in local extinctions. Modified from[1].

Exotic species	Invaded system	Description
Oxalis pes-caprae (annual geophyte)	Mediterrannean islands	Replaced vulnerable and endemic ruderal species[30].
Ageratina adenophora (shrub)	Subtropics of India and China	Reduces local native species richness in introduced range in India and China, whereas no effect on native richness in native range of Mexico[31]. In 25 m^2 plots in China, there were, on average, >25 native species per plot when *A. adenophora* cover was near zero but native species richness declined to <5 species per plot when *A.adenophora* cover was >60%[32].
Chrysanthemoides monilifera subsp. *rotundata* (shrub)	Dunes, eastern Australia	Native species richness reduced by 35–75% across a range of life forms in invaded sites[33].
Cinchona pubescens (tree)	Uplands, Santa Cruz, Galápagos	Over 50% decline in diversity and cover, but not richness, of native species[34].

Imperata cylindrica (rhizomatous grass)	Longleaf pine flatwoods, Mississippi, USA	Reduced plot species richness by 60-80% within 3 years; by increasing shade, can displace shorter plants (i.e. most groundcover herbs)[35].
Agropyron cristatum (perennial grass)	North American prairie	Invades native prairie from abandoned agricultural fields; at 30% cover, reduces overall diversity by 35% (gamma and beta diversity both affected)[36].
Taeniatherum caput-medusae (annual grass)	North American sagebrush	Plant diversity in heavily invaded sites reduced by 85% and richness by 60% compared with uninvaded sites[37].
Microstegium vimineum (annual grass)	Forest understoreys, USA	Reduced native plant species diversity by 38% within two years[13,14].
Fallopia spp. (rhizomatous forbs)	Europe	Reduce species diversity and alter community structure[38].
Tradescantia fluminensis (clonal forb)	Forest understoreys, New Zealand	10-fold reduction in tree seedling species richness in forest understoreys in invaded sites compared with uninvaded sites[39].
Ligustrum robustum subsp. *walkeri* (shrub)	La Réunion wet forests	At least 5 native species threatened with extinction[11].

Lonicera tatarica (shrubby honeysuckle)	Woodlands, northeastern USA	In mesic stand with rich soil, herb cover and species richness and tree seedling density substantially depressed when *L. tatarica* cover > 30%[40].
Miconia calvescens (tree)	Rainforests, Tahiti	Reduced fecundity of understorey trees; forms monotypic stands; directly threatens 40-50 species (almost 50% of Tahiti's endemic flora), which are on the verge of extinction[12].
Ruellia simplex (perennial forb)	Floodplain forests, southern USA	Propagules from urban gardens supplement the local seed supply, creating and maintaining monocultures that displace native plant species[41].
Stenotaphrum secundatum (perennial grass)	Coastal swamp forest, eastern Australia	Local extinctions of native plant species (83% fewer native species) and an 85% reduction in rates of woody plant recruitment in invaded sites[42].

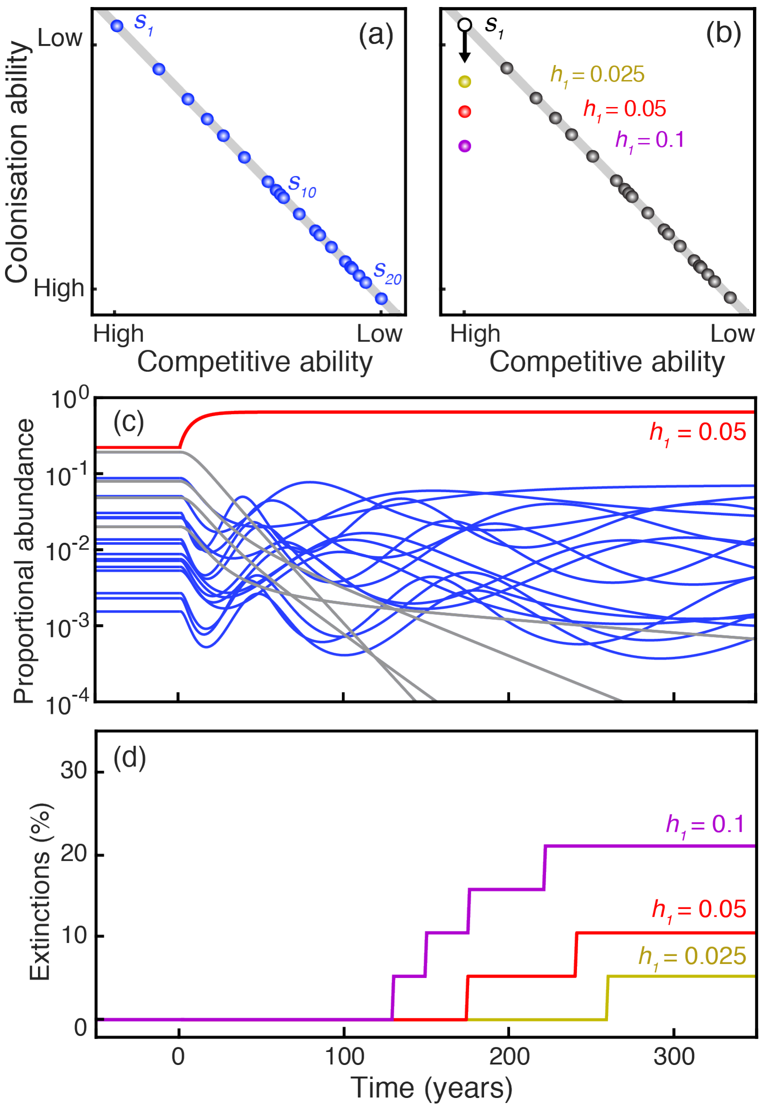

Supplementary Figure 1: Effects of adding external colonists of the superior competitor, s_1, to the same community shown in Fig. 1 in main paper. a) Original and b) disrupted tradeoff surface, highlighting that all 19 native species (grey circles) could potentially be displaced by s_1, but increases in h_1 of 0.025, 0.05 and 0.1 only displace 1, 2 and 4 native species respectively; c) relative abundance of the 20 species after elevating h_1 to 0.05; d) a timeline of extinctions as a consequence of elevating h_1 to 0.025, 0.05 and 0.1. Details as in Fig. 1. Because of the distribution of species along the tradeoff surface in this community (i.e. where s_1 is more ecologically dissimilar to its inferior competitors than s_{10} is to its inferior competitors), increases in h_1 of up to 0.1 resulted in fewer extinctions than equivalent increases in h_{10} (cf. Fig. 1 in main paper).

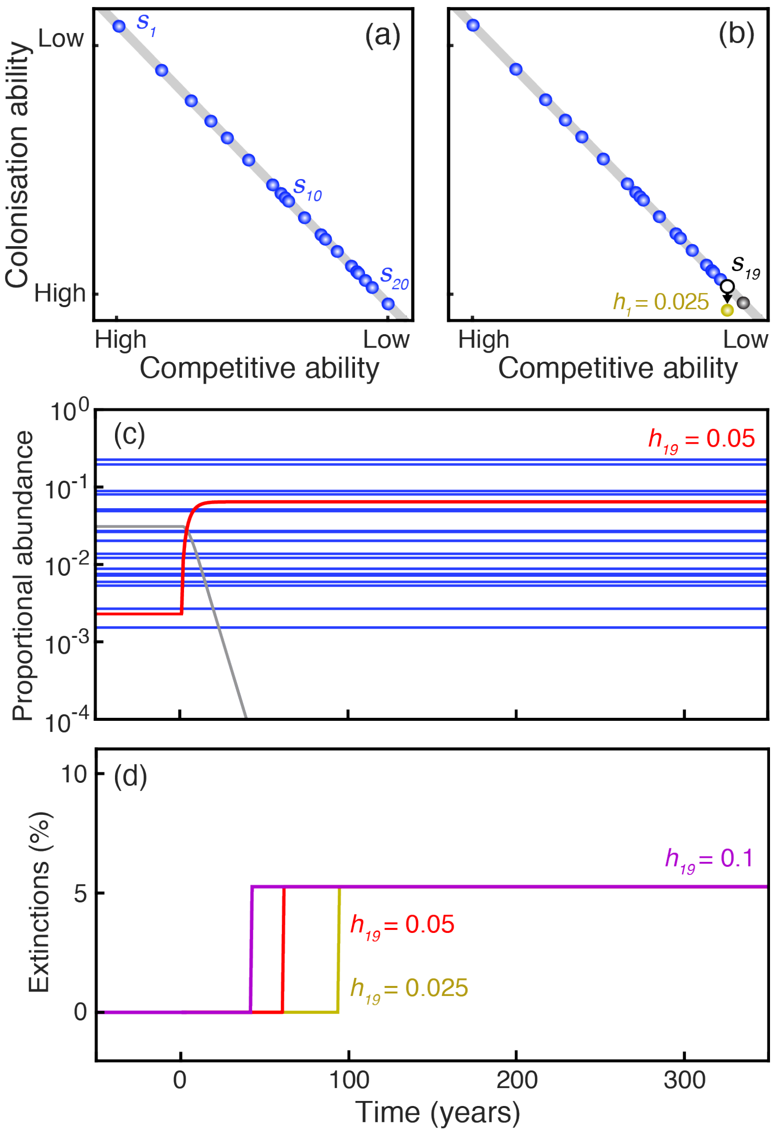

Supplementary Figure 2: Effects of adding external colonists of the second worst competitor, s_{19}, to the same community shown in Fig. 1 in main paper. a) Original and b) disrupted tradeoff surface, highlighting that only 1 native species could potentially be displaced by s_{19} regardless of the number of external colonists added (i.e. value of h_{19}); c) Relative abundance of the 20 species after elevating h_{19} to 0.05; d) a timeline of extinctions as a consequence of elevating h_{19} to 0.025, 0.05 and 0.1, showing differences in extinction rate but not magnitude. Details as in Fig. 1; only the shifted position of s_{19} when h_{19} = 0.025 is shown in (b).

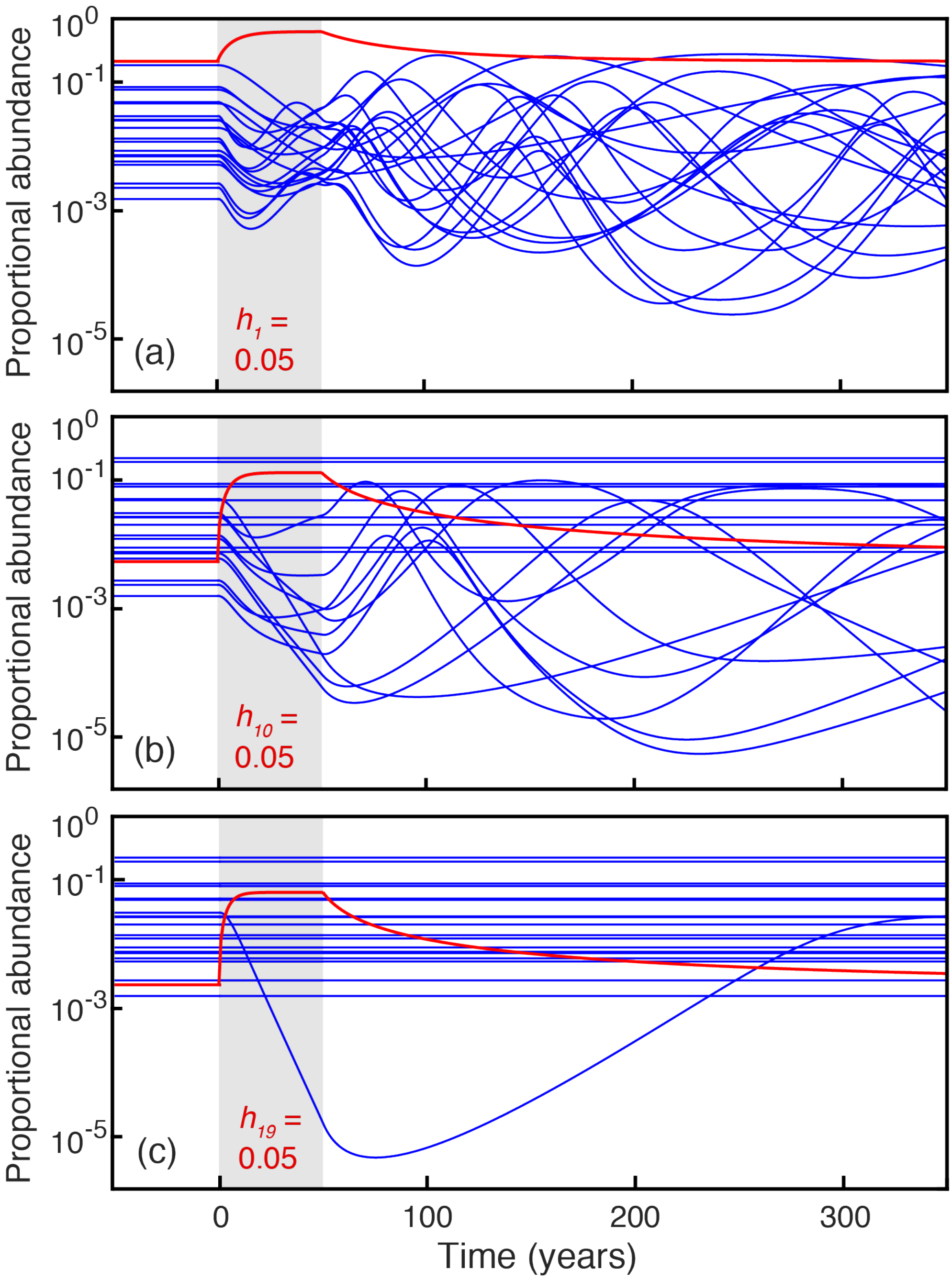

Supplementary Figure 3: Relative abundance of the 20 species in the same community as shown in Fig. 1 in main paper after temporarily elevating h_i by 0.05 of a) s_1, b) s_{10} and c) s_{19} by 0.1 for 50 years (shaded region). This figure illustrates the effects of temporary, as opposed to permanent (cf. Fig. 1, Supplementary Figs. 1-2), increases in h_i.

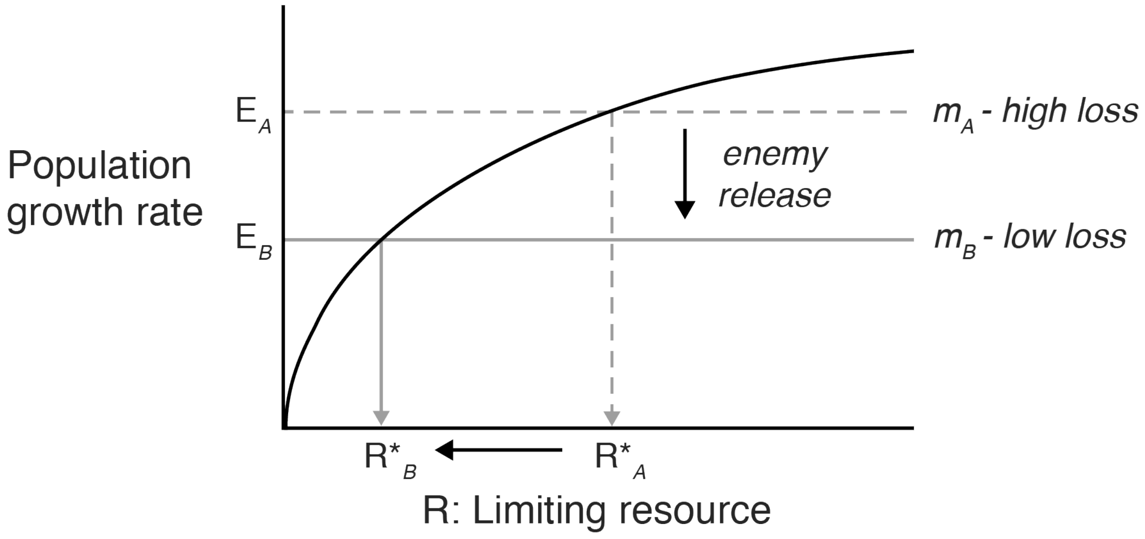

Supplementary Figure 4: Relationship between population growth rate of a single species relative to the availability of a limiting resource showing how the extent of biomass loss (horizontal grey lines, m_A cf m_B) affects a species' competitive ability (known as R*[43]). The growth rate of a species' population abundance is determined by rates of per capita growth (solid black surface) and tissue loss or mortality (horizontal grey lines) relative to a limiting resource (R). A population is at equilibrium growth rate (E_A, E_B) when its growth and loss are equal, and the associated resource level indicates a species' R* (arrows pointing at R), the minimum amount of resource required for the species' persistence. Lower R* values indicate higher competitive ability. When tissue loss is reduced because of enemy release (m_A to m_B), a population can attain positive growth rates at lower levels of resource availability without changes in its intrinsic per capita growth rate. Such changes reduce a species' R* (R^*_A to R^*_B) and thus increase its competitive ability. Adapted from [43] and [25].

Supplementary References

1 Catford, J. A. *et al.* The intermediate disturbance hypothesis and plant invasions: Implications for species richness and management. *Perspect. Plant Ecol. Evol. Systemat.* **14,** 231-241 (2012).

2 Salguero-Gómez, R. Applications of the fast–slow continuum and reproductive strategy framework of plant life histories. *New Phytologist* **213,** 1618-1624 (2017).

3 Molina-Montenegro, M. A., Cleland, E. E., Watts, S. M. & Broitman, B. R. Can a breakdown in competition–colonization tradeoffs help explain the success of exotic species in the California flora? *Oikos* **121,** 389–395 (2012).

4 Jo, I., Fridley, J. D. & Frank, D. A. Linking above- and belowground resource use strategies for native and invasive species of temperate deciduous forests. *Biol. Invasions* **17,** 1545-1554 (2015).

5 Fridley, J. D. Extended leaf phenology and the autumn niche in deciduous forest invasions. *Nature* **485,** 359 (2012).

6 Fridley, J. D. & Craddock, A. Contrasting growth phenology of native and invasive forest shrubs mediated by genome size. *New Phyt.* **207,** 659-668 (2015).

7 Adams, J., Fang, W., Callaway, R., Cipollini, D. & Newell, E. A cross-continental test of the Enemy Release Hypothesis: leaf herbivory on *Acer platanoides* (L.) is three times lower in North America than in its native Europe. *Biol. Invasions* **11,** 1005-1016 (2009).

8 Martin, P. H., Canham, C. D. & Kobe, R. K. Divergence from the growth–survival trade-off and extreme high growth rates drive patterns of exotic tree invasions in closed-canopy forests. *J. Ecol.* **98,** 778-789 (2010).

9 Feng, Y.-L. *et al.* A quicker return energy-use strategy by populations of a subtropical invader in the non-native range: a potential mechanism for the evolution of increased competitive ability. *J. Ecol.* **99,** 1116-1123 (2011).

10 DeWalt, S. J., Denslow, J. S. & Ickes, K. Natural enemy release facilitates habitat expansion of the invasive tropical shrub *Clidemia hirta*. *Ecology* **85,** 471-483 (2004).

11 Lavergne, C., Rameau, J.-C. & Figier, J. The invasive woody weed *Ligustrum robustum* subsp. *walkeri* threatens native forests on La Réunion. *Biol. Invasions* **1,** 377-392 (1999).

12 Meyer, J.-Y. & Florence, J. Tahiti's native flora endangered by the invasion of *Miconia calvescens* DC. (Melastomataceae). *J. Biogeog.* **23,** 775-781 (1996).

13 Flory, S. L. & Clay, K. Non-native grass invasion alters native plant composition in experimental communities. *Biol. Invasions* **12,** 1285-1294 (2010).

14 Eschtruth, A. K. & Battles, J. J. Assessing the relative importance of disturbance, herbivory, diversity, and propagule pressure in exotic plant invasion. *Ecol. Mono.* **79,** 265-280 (2009).

15 Bellingham, P. J., Tanner, E. V. J. & Healey, J. R. Hurricane disturbance accelerates invasion by the alien tree *Pittosporum undulatum* in Jamaican montane rain forests. *J. Veg. Sci.* **16,** 675-684 (2005).

16 Joshi, J. & Vrieling, K. The enemy release and EICA hypothesis revisited: incorporating the fundamental difference between specialist and generalist herbivores. *Ecol. Lett.* **8,** 704-714 (2005).

17 Kitajima, K., Fox, A. M., Sato, T. & Nagamatsu, D. Cultivar selection prior to introduction may increase invasiveness: evidence from *Ardisia crenata*. *Biol. Invasions* **8,** 1471-1482 (2006).

18 Driscoll, D. A. *et al.* New pasture plants intensify invasive species risk. *Proc. Natl Acad. Sci.* **111,** 16622–16627 (2014).

19 Chrobock, T., Kempel, A., Fischer, M. & van Kleunen, M. Introduction bias: Cultivated alien plant species germinate faster and more abundantly than native species in Switzerland. *Basic Appl. Ecol.* **12,** 244-250 (2011).

20 Buckley, Y. M. & Catford, J. Does the biogeographic origin of species matter? Ecological effects of native and non-native species and the use of origin to guide management. *J. Ecol.* **104,** 4-17 (2016).

21 Calcagno, V., Mouquet, N., Jarne, P. & David, P. Coexistence in a metacommunity: the competition–colonization trade-off is not dead. *Ecol. Lett.* **9,** 897-907 (2006).

22 Arismendi, I. *et al.* Aquaculture, non-native salmonid invasions and associated declines of native fishes in Northern Patagonian lakes. *Freshwater Biol.* **54,** 1135-1147 (2009).

23 Wilson, J. R. U., Dormontt, E. E., Prentis, P. J., Lowe, A. J. & Richardson, D. M. Something in the way you move: dispersal pathways affect invasion success. *Trends Ecol. Evol.* **24,** 136-144 (2009).

24 Tilman, D., May, R. M., Lehman, C. L. & Nowak, M. A. Habitat destruction and the extinction debt. *Nature* **371,** 65-66 (1994).

25 Louda, S. M., Keeler, K. H. & Holt, R. D. in *Perspectives on Plant Competition* (eds J.B. Grace & D. Tilman) 413-444 (Academic Press, San Diego, 1990).

26 Callaway, R. M. & Ridenour, W. M. Novel weapons: invasive success and the evolution of increased competitive ability. *Front. Ecol. Environ.* **2,** 436-443 (2004).

27 Bais, H. P., Vepachedu, R., Gilroy, S., Callaway, R. M. & Vivanco, J. M. Allelopathy and exotic plant invasion: from molecules and genes to species interactions. *Science* **301,** 1377-1380 (2003).

28 Harpole, W. S. & Tilman, D. Grassland species loss resulting from reduced niche dimension. *Nature* **446,** 791-793 (2007).

29 Harpole, W. S. *et al.* Addition of multiple limiting resources reduces grassland diversity. *Nature* **537,** 93-96 (2016).

30 Vilà, M. *et al.* Local and regional assessments of the impacts of plant invaders on vegetation structure and soil properties of Mediterranean islands. *J. Biogeog.* **33,** 853-861 (2006).

31 Inderjit *et al.* Volatile chemicals from leaf litter are associated with invasiveness of a Neotropical weed in Asia. *Ecology* **92,** 316-324 (2011).

32 Lu, Z. & Ma, K. Scale dependent relationships between native plant diversity and the invasion of croftonweed (Eupatorium adenophorum) in southwest China. *Weed Sci.* **53,** 600-604 (2017).

33 Mason, T. J. & French, K. Impacts of a woody invader vary in different vegetation communities. *Divers. Distrib.* **14,** 829-838 (2008).

34 Jäger, H., Kowarik, I. & Tye, A. Destruction without extinction: long-term impacts of an invasive tree species on Galápagos highland vegetation. *J. Ecol.* **97,** 1252-1263 (2009).

35 Brewer, S. Declines in plant species richness and endemic plant species in longleaf pine savannas invaded by *Imperata cylindrica*. *Biol. Invasions* **10,** 1257-1264 (2008).

36 Heidinga, L. & Wilson, S. D. The impact of an invading alien grass (Agropyron cristatum) on species turnover in native prairie. *Divers. Distrib.* **8,** 249-258 (2002).

37 Davies, K. Plant community diversity and native plant abundance decline with increasing abundance of an exotic annual grass. *Oecologia* **167,** 481-491 (2011).

38 Hejda, M., Pyšek, P. & Jarošík, V. Impact of invasive plants on the species richness, diversity and composition of invaded communities. *J. Ecol.* **97,** 393-403 (2009).

39 Standish, R. J., Williams, P. A. & Robertson, A. W. The impact of an invasive weed *Tradescantia fluminensis* on native forest regeneration. *J. Appl. Ecol.* **38,** 1253-1263 (2001).

40 Woods, K. D. Effects of Invasion by *Lonicera tatarica* L. on Herbs and Tree Seedlings in Four New England Forests. *Am. Mid. Nat.* **130,** 62-74 (1993).

41 Smith, A. M., Reinhardt Adams, C., Wiese, C. & Wilson, S. B. Re-vegetation with native species does not control the invasive *Ruellia simplex* in a floodplain forest in Florida, USA. *Appl. Veg. Sci.* **19,** 20-30 (2016).

42 Gooden, B. & French, K. Non-interactive effects of plant invasion and landscape modification on native communities. *Divers. Distrib.* **20,** 626-639 (2014).

43 Tilman, D. *Resource Competition and Community Structure*. (Princeton University Press, Princeton, 1982).
